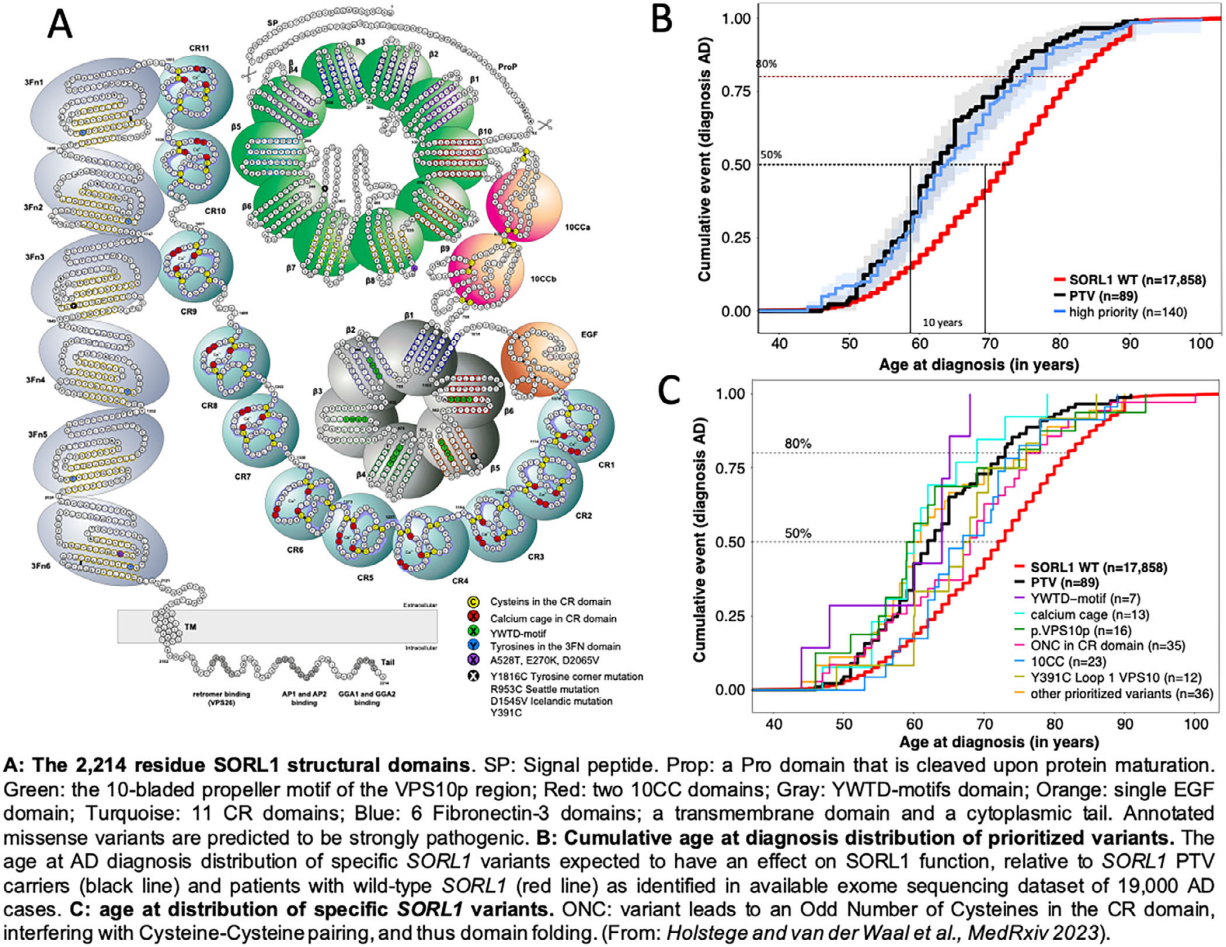# The effect of specific *SORL1* variants on Alzheimer’s Disease risk supports the existence of highly penetrant *SORL1* variants

**DOI:** 10.1002/alz.084836

**Published:** 2025-01-03

**Authors:** Henne Holstege, Matthijs W J de Waal, Niccoló Tesi, Sven J van der Lee, Maartje Vogel, Rosalina M.L. van Spaendonk, Marc Hulsman, Olav M Andersen, ADES‐consortium, ADSP consortium, StEP‐AD consortium, Knight‐ADRC, UCSF/NYGC/UAB

**Affiliations:** ^1^ Amsterdam UMC, Amsterdam Netherlands; ^2^ Aarhus University, Aarhus Denmark

## Abstract

**Background:**

The sortilin‐related receptor 1 protein, SORL1, interacts with retromer to regulate trafficking of cargo out of the early endosome. Genetic variants in *SORL1* that lead to a premature protein truncation (PTVs) are observed almost exclusively in Alzheimer’s disease (AD) patients, suggesting *SORL1*’s haploinsufficiency may be causal for AD. However, the large majority of *SORL1* variants are rare missense variants which affect diverse structural domains, some of which may be causative for disease or (strongly) risk‐increasing, while others are (likely) benign.

**Methods:**

We prioritized functionally relevant SORL1 protein‐residues by domain‐mapping of disease mutations, DMDM, (Figure A). We evaluated the effect of prioritized genetic *SORL1*‐variants identified in 18,959 AD cases and 21,893 non‐demented controls, on age at onset and on disease risk. Second, since functional SORLA is cleaved at the neuronal membrane into soluble SORLA (sSORLA) we used Western Blot and proteomics to test whether *SORL1* variants affected the level of sSORLA in CSF.

**Results:**

Prioritized missense variants (PMVs) associated with a 10.5‐fold increased risk of early onset AD (p = 3.0 × 10^‐29^) and a 4.5‐fold increased risk of late onset AD (p = 4.9 × 10^‐11^). The distribution of the ages at onset of PMV‐ and PTV‐carriers overlapped (Figure B), which was on average >8‐years earlier than carriers of wild‐type *SORL1*, regardless of *APOE* genotype. Variants affecting the ‘YWTD‐motif’ or ‘calcium cage’ residues (Figure A) were observed only in AD cases. Carriers of such variants had an *earlier* age at onset compared to carriers of PTVs, indicative of a dominant negative effect, while other variants had a milder effect than losing a copy (Figure C). Using WB, we found that sSORLA levels in carriers of specific PMVs were reduced to ∼50% relative to carriers of benign *SORL1* variants, while levels of PTVs were reduced to ∼65%.

**Discussion:**

Specific SORL1 variants occur only in AD cases and have an early age at onset, and some may have a dominant negative effect, independent of *APOE* background. Carriers of these variants might be identified by a very low level of CSF sSORLA. Such variants might be grouped with pathogenic variants in the three autosomal dominant AD genes *PSEN1*/*2* and *APP*.